# Evaluation of a SARS-CoV-2 Capture IgM Antibody Assay in Convalescent Sera

**DOI:** 10.1128/Spectrum.00458-21

**Published:** 2021-09-08

**Authors:** Binh Ha, Samadhan Jadhao, Laila Hussaini, Theda Gibson, Kathy Stephens, Luis Salazar, Caroline Ciric, Meg Taylor, Nadine Rouphael, Srilatha Edupuganti, Christina A. Rostad, S. Mark Tompkins, Evan J. Anderson, Larry J. Anderson

**Affiliations:** a Division of Pediatric Infectious Diseases, Emory University School of Medicine and Children’s Health Care of Atlanta, Atlanta, Georgia, USA; b Division of Infectious Diseases, Department of Medicine, Emory University School of Medicine, Atlanta, Georgia, USA; c Department of Infectious Diseases, University of Georgia, Athens, Georgia, USA; d Center for Vaccines and Immunology, University of Georgia, Athens, Georgia, USA; e Emory-UGA Centers of Excellence for Influenza Research and Surveillance (CEIRS), Athens, Georgia, USA; University of Mississippi Medical Center.

**Keywords:** SARS-CoV-2, COVID-19, capture IgM ELISA, IgG ELISA, antibody duration

## Abstract

Severe acute respiratory syndrome coronavirus 2 (SARS-CoV-2) is responsible for a global pandemic with over 152 million cases and 3.19 million deaths reported by early May 2021. Understanding the serological response to SARS-CoV-2 is critical to determining the burden of infection and disease (coronavirus disease 2019 [COVID-19]) and transmission dynamics. We developed a capture IgM assay because it should have better sensitivity and specificity than the commonly used indirect assay. Here, we report the development and performance of a capture IgM enzyme-linked immunosorbent assay (ELISA) and a companion indirect IgG ELISA for the spike (S) and nucleocapsid (N) proteins and the receptor-binding domain (RBD) of S. We found that among the IgM ELISAs, the S ELISA was positive in 76% of 55 serum samples from SARS-CoV-2 PCR-positive patients, the RBD ELISA was positive in 55% of samples, and the N ELISA was positive in 15% of samples. The companion indirect IgG ELISAs were positive for S in 89% of the 55 serum samples, RBD in 78%, and N in 85%. While the specificities for IgM RBD, S, and N ELISAs and IgG S and RBD ELISAs were 97% to 100%, the specificity of the N IgG ELISA was lower (89%). RBD-specific IgM antibodies became undetectable by 3 to 6 months, and S IgM reached low levels at 6 months. The corresponding IgG S, RBD, and N antibodies persisted with some decreases in levels over this time period. These capture IgM ELISAs and the companion indirect IgG ELISAs should enhance serologic studies of SARS-CoV-2 infections.

**IMPORTANCE** Severe acute respiratory syndrome coronavirus 2 (SARS-CoV-2) has inflicted tremendous loss of lives, overwhelmed health care systems, and disrupted all aspects of life worldwide since its emergence in Wuhan, China, in December 2019. Detecting current and past infection by PCR or serology is important to understanding and controlling SARS-CoV-2. With increasing prevalence of past infection or vaccination, IgG antibodies are less helpful in diagnosing a current infection. IgM antibodies indicate a more recent infection and can supplement PCR diagnosis. We report an alternative method, capture IgM, to detect serum IgM antibodies, which should be more sensitive and specific than most currently used methods. We describe this capture IgM assay and a companion indirect IgG assay for the SARS-CoV-2 spike (S), nucleocapsid (N), and receptor-binding domain (RBD) proteins. These assays can add value to diagnostic and serologic studies of coronavirus disease 2019 (COVID-19).

## INTRODUCTION

Severe acute respiratory syndrome coronavirus 2 (SARS-CoV-2) is member of the *Betacoronavirus* genus and *Coronaviridae* family (1) that includes the original severe acute respiratory syndrome coronavirus (SARS-CoV), which emerged in 2003 (2). SARS-CoV-2, which was first reported in Wuhan, China, in December 2019 (3), causes the severe respiratory disease coronavirus disease 2019 (COVID-19). As of early May 2021, over 152 million confirmed cases and 3.19 million deaths were reported to WHO (4). SARS-CoV-2 prompted an unparalleled research effort to characterize the virus, its infection, epidemiology, disease pathogenesis, transmission, and control and to develop treatments and vaccines. Many features of SARS-CoV-2 and COVID-19 have now been described, but much is left to learn (5–9).

Critical to understanding SARS-CoV-2 infections and epidemiology is diagnosing acute and prior infection. Acute infection is most often detected with molecular detection of nucleic acid. Serologic studies for SARS-CoV-2 (10–13) antibodies are primarily used to document prior infection, with many described in the literature (14). In most infections, kinetics of the antibody responses demonstrate an early IgM response followed closely by an IgG response. The commonly used indirect IgM assays may lose sensitivity as IgG antibodies increase and outcompete IgM antibodies and give false-positive results in specimens with rheumatoid factor (15, 16). In this report, we describe a capture IgM antibody assay specific for the spike (S) protein, the nucleocapsid (N) protein, and the receptor-binding domain (RBD) of the S protein of SARS-CoV-2. The capture IgM assay format minimizes the potential for IgG to block detection of IgM and for rheumatoid factor to give false-positive results and could enhance serologic studies of COVID-19. Since prior studies of SARS-CoV and SARS-CoV-2 show frequent (often greater than 90%) detection of antibody responses to S and N proteins and RBD after documented infection (17–21), we chose to evaluate all three of these antigens in our IgM and IgG antibody assays.

## RESULTS

**FIG 1:**
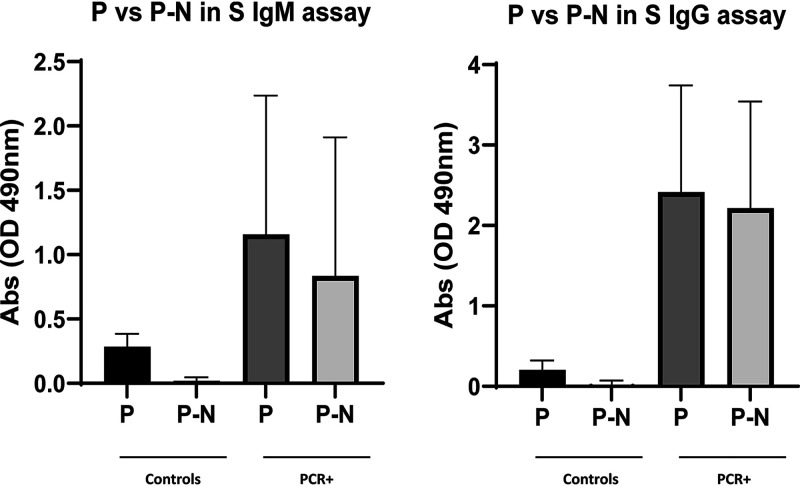
Comparison of the separation of P and P-N absorbance values between controls and PCR^+^ specimens for S IgM and IgG ELISAs. Controls are serum specimens collected before 2020 (*n* = 113; 40 pairs and 33 single serum samples), and PCR^+^ samples are 55 serum samples from 55 patients with PCR^+^ COVID-19. P is the average absorbance (2 wells/specimen) against S antigen, and P-N is P minus the average absorbance (2 wells/specimen) against control antigen. The graph shows mean values and standard deviations. Note the much clearer separation between control and PCR^+^ specimens for P-N than for P.

After optimizing assay conditions, we considered the value of including control antigen wells for each specimen in the IgM assay. We found considerable variations in the specimen-specific absorbance for control antigen (293F cell lysate), ranging from 0.18 to 1.2 absorbance units with all but one between 0.18 and 0.72 absorbance units. The P-N value (P is the absorbance against the test antigen and N is the absorbance against the control antigen) gave better separation between cases and controls with IgM and IgG enzyme-linked immunosorbent assays (ELISAs) (Fig. 1). Consequently, we decided to include control antigen wells for each patient and use the P-N value for analysis.

We next determined the sensitivity and specificity of the three IgM and IgG assays (with RBD, S, and N antigens) by testing their performance with 55 serum samples from PCR^+^ patients and 113 serum samples from control patients. The PCR^+^ patient serum samples were collected between 25 and 138 days post-symptom onset (PSO), with a mean of 54 days. The control serum samples were used to calculate cutoff values for the assays and to determine assay specificity. We considered specimens with P-N values greater than the mean plus 3 standard deviations of the P-N values of control specimens as positives. The cutoff P-N values for the IgM ELISAs were 0.101, 0.060, and 0.100 for S, RBD, and N, respectively. With these cutoffs, one control specimen was positive for S IgM and one was positive for RBD IgM, yielding a specificity of 112/113 (99%) for each; three specimens were positive for N IgM, giving a specificity of 110/113 (97%). Two of these three specimens were an acute-convalescent pair from a rhinovirus PCR^+^ patient with no significant change in P-N values between the paired specimens. Also, none of the IgM-positive specimens were IgG positive.

Because previous studies have shown some persons who were never exposed to SARS-CoV-2 have IgG antibodies induced by other coronaviruses (22), we decided to exclude negative-control specimens with high outlier P-N values from our calculation of IgG cutoff values. We considered these serum specimens to have cross-reacting antibodies specific to SARS-CoV-2 and, therefore, were not true negative-control specimens (Fig. 1; Fig. S1 in the supplemental material). Specifically, we considered 10 specimens to have outlier P-N values for N protein (P-N = 0.195, 0.203, 0.203, 0.216, 0.305, 0.334, 0.404, 0.42, 1.361, and 1.547), 2 specimens to have outlier P-N values for S protein (P-N = 0.212 and 0.419), and 0 specimens to have outlier P-N values for RBD, and we excluded these specimens from the cutoff determination. The cutoff values for the IgG ELISAs were 0.114, 0.090, and 0.177 for S, RBD, and N, respectively. With these cutoff values, two control specimens were positive for S IgG antibodies, 0 were positive for RBD IgG antibodies, and 12 specimens from 8 patients were positive for N IgG antibodies. The two patients with the S-positive serum specimens had a respiratory panel PCR-negative illness. Among the 12 specimens positive for N IgG antibodies, 8 were from 4 acute- and convalescent-phase serum pairs (one each from common endemic coronavirus [229E, OC43, and NL63] PCR^+^ patients and one from an influenza A PCR^+^ patient). There were no significant differences in P-N values between these paired specimens. The other four serum specimens were from patients without detectable virus.

**FIG 2:**
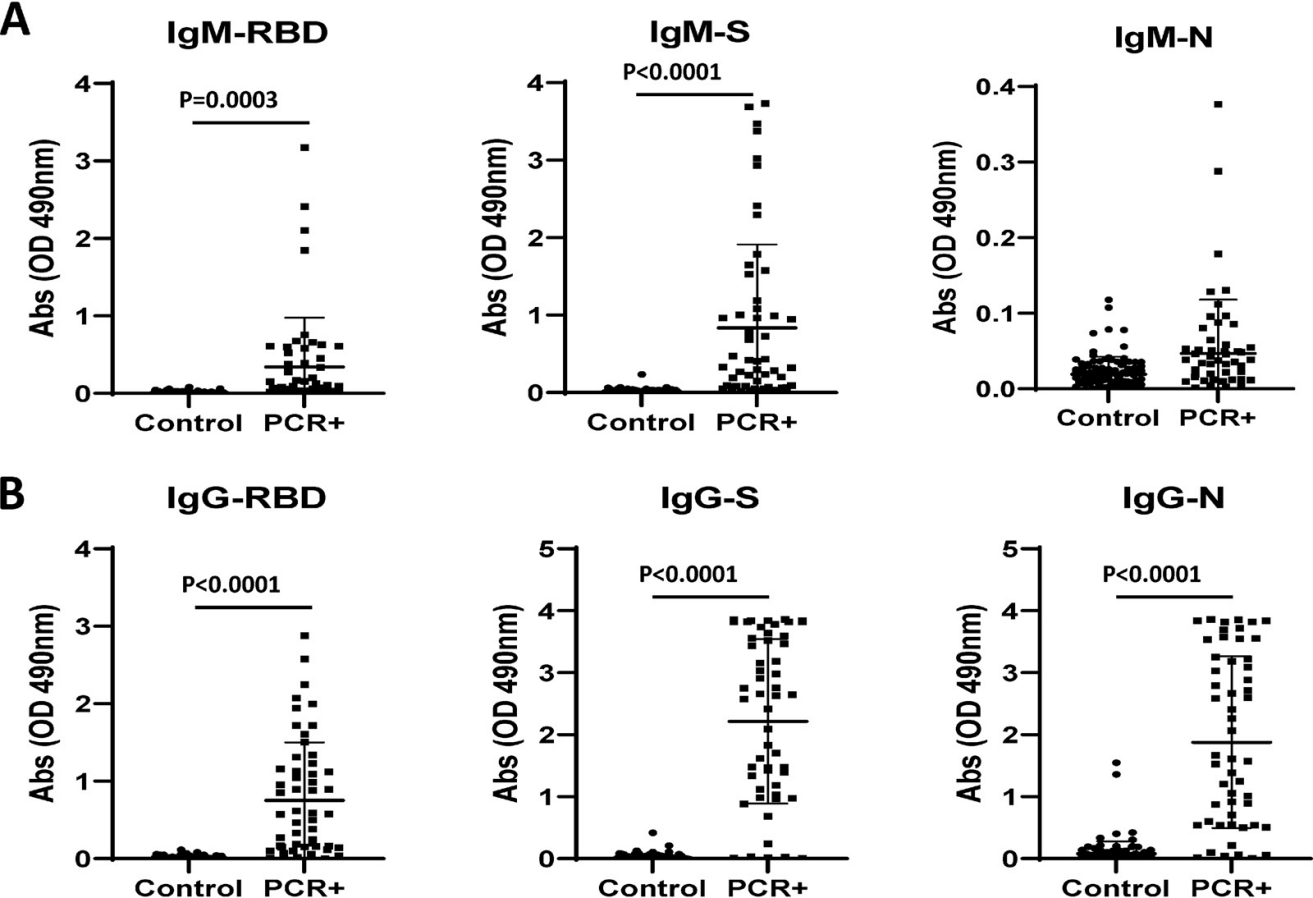
COVID-19 patient serum samples possess SARS-CoV-2-specific IgG and IgM antibodies. The P-N absorbance values for control specimens, serum samples collected before 2020, and PCR^+^ specimens, 55 serum samples from 55 patients with PCR^+^ COVID-19, are depicted. Specimens were tested at a 1:200 dilution against receptor-binding domain (RBD), spike protein (S), or nucleocapsid protein (N) antigens. Controls (*n* = 73; 40 pairs and 33 nonpaired serum samples) were from healthy non-COVID-19 adults with and without other respiratory infections. Data points are P-N values, and horizontal lines indicate mean and mean + standard deviation (SD). (A) The absorbance values of controls and COVID-19 specimens are shown in IgM ELISAs with RBD-His, S-His, and N-His as antigens. Horizonal lines indicate mean absorbance values. The cutoff values for RBD, S, and N are 0.060, 0.101, and 0.100, respectively. (B) The absorbance values of controls and COVID-19 specimens are shown in IgG ELISAs with RBD-His, S-His, and N-His as antigens. Horizontal lines indicate mean absorbance values. The cutoff values for RBD, S, and N are 0.114, 0.090, and 0.177, respectively. Note the difference in scale for the IgM-N assay.

Moreover, the specificity of the different IgG ELISAs was 111/113 (98%), 113/113 (100%), and 101/113 (89%) for S, RBD, and N, respectively. Among the 55 serum samples from SARS-CoV-2 PCR-positive patients, 42 were positive for S IgM antibodies (sensitivity of 76%), 30 were positive for RBD IgM antibodies (sensitivity of 55%), and 8 were positive for N IgM antibodies (sensitivity of 15%). In comparison, we detected S IgG antibodies in 49 samples (sensitivity of 89%), RBD IgG antibodies in 43 samples (sensitivity of 78%), and N IgG antibodies in 47 samples (sensitivity of 85%) (Fig. 2A and B). The correlation between specificity and sensitivity of both IgM and IgG assays for all antigens are shown in Fig. S2A and B. Also, the number of specimens positive for more than one antigen are listed in Table 1. The infrequent detection of IgM N antibodies with our assay was surprising but consistent with a prior study that identified no significant differences in N-specific IgM antibodies between healthy donors and recovered COVID-19 patients (23) and with another study that showed similar low levels of N-specific IgM antibody detection between COVID-19 patients and controls (20). Among the suspected COVID-19 cases (the remaining 26 from 81 patients), one specimen had S-, RBD-, and N-specific IgM and IgG antibodies, one contained only RBD-specific IgM antibodies, two had only S- and N-specific IgG antibodies, two had only S-specific IgG antibodies, and two had only N-specific IgG antibodies. The disparities in IgG and IgM antibody detection are likely explained by the time during the illness at which the serum specimens were collected (i.e., the IgM-positive serum specimen may have been collected earlier in the illness, and the IgG-positive serum specimen may have been collected later in the illness).

**TABLE 1:** Summary of COVID-19 PCR^+^ specimens from IgG and IgM ELISAs demonstrating the concordance of IgG and IgM antibody results for 55 COVID-19 PCR^+^ patients

IgM ELISA				IgG ELISA			
All^+^^*a*^	S^+^			All^+^^*a*^	S^+^		
	RBD^+^N^−^	RBD^−^N^+^	RBD^−^N^−^		RBD^+^N^−^	RBD^−^N^+^	RBD^−^N^−^
8 (15%)	20 (36%)	1 (2%)	7 (13%)	43 (78%)	1 (2%)	3 (5%)	2 (4%)

a S^+^, RBD^+^ and N^+^ indicate antibody positive and S^−^, RBD^−^, and N^−^ indicate antibody negative. All^+^, indicates antibody positive for S, RBD, and N. Results shown are the numbers of positive specimens in their respective subgroups and the percentage from the total number.

**FIG 3:**
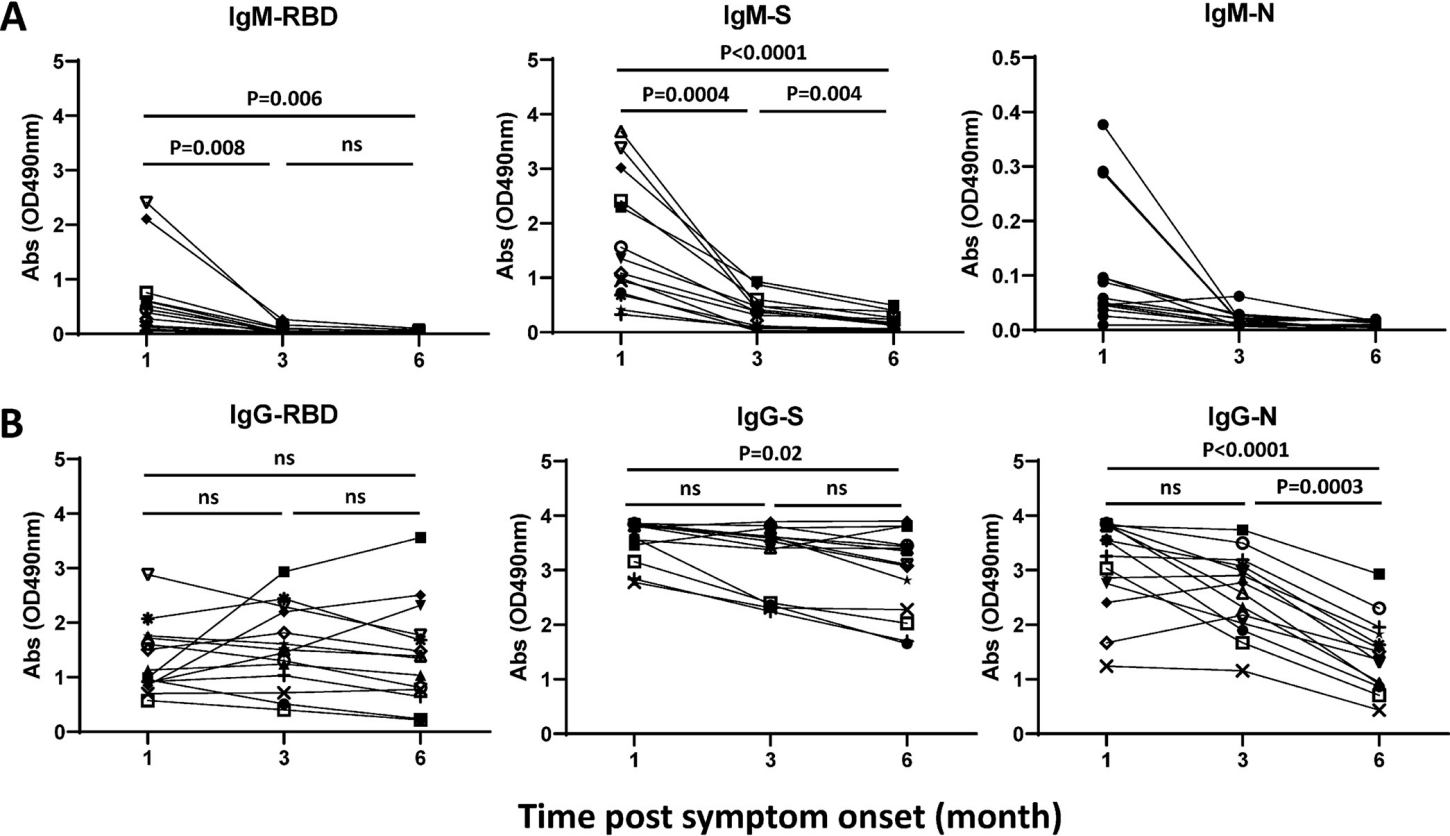
Longitudinal analysis of SARS-CoV-2-specific IgM and IgG antibodies in COVID-19 patients. Data points are IgM and IgG P-N values for serial specimens at 1, 3, and 6 months PSO from 14 COVID-19 PCR^+^ patients. The three serum samples from each patient were run at the same time. (A) The absorbance values of COVID-19 specimens (*n* = 14) collected at 1, 3, and 6 months PSO are shown in IgM ELISAs using RBD, S, and N antigens. The cutoff values for RBD, S, and N are 0.060, 0.101, and 0.100, respectively. (B) The absorbance values of COVID-19 specimens (*n* = 14) collected at 1, 3, and 6 months PSO are shown in IgG ELISAs using RBD, S, and N antigens; ns, not significant. The cutoff values for RBD, S, and N are 0.114, 0.090, and 0.177, respectively. Note the difference in scale for the IgM-N assay.

A decrease in infection-induced SARS-CoV-2 serum antibody levels over time has been described previously (24–26). In our sample of serial serum specimens from 14 PCR^+^ patients, we observed a significant reduction in S- and RBD-specific IgM and N-specific IgG antibody levels with increasing time after symptom onset. In this analysis, our data show a rapid decline of RBD-specific IgM antibodies between 1 and 3 months, with many specimens reaching baseline levels at 3 months PSO (10 of 14 specimens became negative). The decline in S-specific IgM antibodies was slower, with many patients still having detectable antibody levels at 6 months (Fig. 3A). The levels of RBD- and S-specific IgG antibody levels fluctuated over time, with the only significant decrease being between 1 and 6 months for S-specific IgG antibodies (*P* = 0.02) (Fig. 3B, middle panel). Actually, relative stability of IgG antibodies through 6 months after illness onset has been described in other studies (27, 28). To further understand the effect of time on the positivity of the IgM assay, we compared the positivity rate for S, RBD, and N antigens in the 55 serum samples from PCR^+^ patients that were collected before 45 days PSO with positivity rates in samples collected 45 or more days PSO. In this analysis, 19/23 (83%), 17/23 (74%), and 6/23 (26%) of serum samples collected before 45 days PSO were positive, while 17/32 (53%), 13/32 (41%), and 3/32 (9%) of serum samples collected 45 or more days PSO were positive for S, RBD, and N IgM antibodies, respectively.

## DISCUSSION

In this study, we report an alternative antibody capture ELISA method for IgM antibodies in serum from COVID-19 patients. This IgM assay has proven reliable for a number of other infectious diseases, such as measles (29), Zika (30), dengue (31), and parvovirus B19 (32). As noted above, previous studies show the capture IgM antibody assay format to have better sensitivity and specificity than the indirect IgM assay format with antigen immobilized on a microtiter plate (15). The potential for false-positive IgM resulting from rheumatoid factor will increase as the prevalence of SARS-CoV-2 IgG antibodies from an earlier infection or vaccination increases. The S protein capture IgM antibody ELISA had a sensitivity of 76% and specificity of 99%, the RBD IgM antibody ELISA had similar specificity (99%) but was less sensitive (55%), and the N IgM antibody ELISA was specific (97%) but not sensitive (15%). The sensitivity of all three IgM ELISAs was higher for serum samples collected before 45 days PSO (i.e., 83% for S, 74% for RBD, and 26% for N). The S IgG ELISA had a sensitivity of 89%, the RBD IgG ELISA had a sensitivity of 78%, and the N IgG ELISA had a sensitivity of 85%. The specificity of the IgG ELISAs was 98%, 100%, and 85% for S, RBD, and N, respectively. The lower specificity for N IgG is consistent with the conservation of N protein amino acid sequences across the coronaviruses (17, 33). One study found a slightly higher percentage of prepandemic serum specimens with IgG antibodies, likely induced by the common cold coronavirus reacting with N (16.2%), S (4.2%), and RBD (0.93%) (22). We are not certain why there was such a low rate of positivity for the N IgM capture ELISA. The increased rate of N IgM positivity in specimens collected less than 45 days PSO indicates that even earlier specimens (e.g., less than 30 days PSO) might give a higher rate of positivity. Unfortunately, we did not have a sufficient number of specimens collected before 30 days PSO to evaluate this possibility. Of note, this low rate of N IgM antibodies is consistent with findings from several other studies (20, 23).

A limitation of these estimates of sensitivity and specificity is the number of serum specimens from PCR^+^ patients (55) and the times at which these specimens were collected (i.e., all were convalescent-phase specimens collected more than 25 days PSO, and 58% were collected between 45 and 138 days PSO). The expected rapid decline in IgM antibodies, as illustrated in our data, results in a substantial decrease in the sensitivity of the IgM assay for the later collected specimens. Serum specimens collected earlier in the illness, including a larger set of serial specimens, would have provided a better indication of the sensitivity of the IgM ELISA.

In addition to the theoretical added sensitivity and specificity of the capture IgM, using a His tag on the protein to detect its capture by IgM antibodies makes it relatively easy to adapt this format to other antigens by replacing one His tag-labeled protein with another and optimizing conditions for this antigen. Note that since we did not compare this capture IgM ELISA to comparable indirect IgM ELISAs, we do not know if this theoretical advantage applies to this capture ELISA for COVID-19 IgM antibodies. Some disadvantages of the capture IgM ELISA include its greater complexity, the additional steps needed to run the assay, and the difficulty in adapting it to rapid testing.

Although the number of serial serum specimens tested is low (*n* = 14), our study did reveal features of IgM and IgG antibody kinetics consistent with other reports. The RBD- and S-specific IgM antibodies declined rapidly over time after symptom onset, with RBD-specific IgM antibodies at baseline at 8 to 10 weeks PSO (data not shown), and S-specific IgM antibodies were at low levels but still detectable at 6 months PSO (Fig. 3). Other studies have demonstrated that IgM antibodies start to decline at approximately 4 weeks PSO (12, 24), and SARS-CoV-2 IgM antibodies decrease more rapidly over time than IgG antibodies (34–36). As noted above, the rapid decline in IgM antibodies did decrease our estimate of the sensitivity of the IgM assay. The rapid decline in IgM antibodies does, however, make a positive IgM indicative of a recent, and not more distant, infection. This ability to serologically differentiate recent from more distant infection in a suspected case without PCR confirmation with IgM is important now because many will have preexisting IgG antibodies. Additionally, the high rates of SARS-CoV-2 IgG in the population substantially increase the risk of a rheumatoid-associated false-positive IgM tests, making minimizing this risk with the capture IgM method of greater value. IgG assays are helpful in assessing response to vaccine and levels of population immunity and, in the case of N IgG, detecting past infection in S protein-vaccinated persons.

In summary, the performance characteristics of the described capture IgM and indirect IgG antibody ELISAs indicate that they can add value to diagnostic and serologic studies of COVID-19.

## MATERIALS AND METHODS

### SARS-CoV-2-infected patients.

Serum samples from 81 patients (55 were PCR^+^) were collected from an acute and convalescent study of outpatients and inpatients with suspected SARS-CoV-2 infection identified between 17 April 2020 and 30 November 2020. Signed patient informed consent was obtained under an institutional review board (IRB)-approved protocol.

### Controls.

We also evaluated 113 serum specimens collected from 73 patients participating in a different IRB-approved study of adults hospitalized with an acute lower respiratory tract illness in 2018 and 2019 prior to the SARS-CoV-2 pandemic. These included 40 acute- and convalescent-phase serum pairs from patients that were BioFire-determined PCR^+^ for endemic coronaviruses (i.e., 229E, NL63, or OC43), respiratory syncytial virus (RSV), rhinovirus, human parainfluenza virus 1 to 4, human metapneumovirus (HMPV), or influenza virus A or B virus. The other 33 serum samples were acute-phase specimens from patients without PCR evidence of a respiratory virus infection.

### SARS-CoV-2 recombinant proteins.

Recombinant SARS-CoV-2 full-length spike and receptor-binding domain (RBD) proteins were produced as described elsewhere (21, 37). Proteins were expressed in Expi293 cells, purified from clarified supernatants by nickel resin chromatography, and dialyzed into phosphate-buffered saline (PBS). The His-tagged N protein was purchased from Sino Biological (Wayne, PA, USA).

### Capture IgM ELISA.

All steps in the ELISA were performed at room temperature (RT). The wash solution, unless otherwise stated, was PBS + 0.05% Tween 20. The capture antibody, 2.5 μg/ml of mouse anti-human IgM antibody (SouthernBiotech, Birmingham, AL, USA) diluted in PBS (Corning, Manassas, VA, USA), was immobilized on a 96-well microtiter plate (Thermo Fisher Scientific, Rochester, NY, USA) overnight at 4°C. The plate was then washed twice with PBS, and blocking buffer (0.33% gelative, 0.33% casein, and 0.33% dry milk dissolved in PBS) was added and incubated for 2 h. The plate was then washed twice, and serum specimens diluted 1:200 in dilution buffer (blocking buffer with 0.15% Tween 20) (BB-T) were added to the plate and incubated for 2 h. The plate was washed 5 times, and SARS-CoV-2 antigen (RBD-His, S-His, N-His, or control antigens) was added to the plate at 1 μg/ml in BB-T and incubated for 2 h. The plate was washed 5 times and incubated with Biotin-conjugated mouse anti-His antibody (0.5 μg/ml in BB-T) (SouthernBiotech) for 1.5 h. The plate was washed 5 times and incubated with horseradish peroxidase (HRP)-conjugated streptavidin at 100 ng/ml (Calbiochem, Temecula, CA, USA) diluted in BB-T and incubated for 1 h. The plate was washed 5 times, and color was developed with *o*-phenylenediamine dihydrochloride (OPD) substrate (Thermo Scientific, Rockford, IL, USA). The reaction was stopped by addition of 4 N H_2_SO_4_, and the absorbance was read at 490 nm using a Synergy H1 plate reader (BioTek, Winooski, VT, USA).

### IgG ELISA.

All steps in the ELISA were performed at RT. The wash solution, unless otherwise stated, was phosphate-buffered saline (PBS) + 0.05% Tween 20. Antigen (RBD-His, S-His, N-His, or control) was diluted in PBS at 0.5 μg/ml (100 μl) and was coated in a 96-well microtiter plate overnight at 4°C. The plate was then washed twice with PBS, and blocking buffer was added and incubated for 2 h. The plate was then washed twice with washing buffer. Serum samples were diluted 1:200 with BB-T and incubated for 1 h 50 min at RT. The plate was then washed 5 times, and HRP-conjugated goat anti-human antibody diluted 1:5,000 in BB-T (Jackson ImmunoResearch, West Grove, PA, USA) was added and incubated for 1 h. Plates were washed 5 times, and the reaction was developed by OPD for 30 min. The reaction was stopped by addition of 4 N H_2_SO_4_, and absorbance was read at 490 nm.

### Statistical analysis.

Analyses were calculated using Prism software (version 9.0). A Wilcoxon signed-rank test and Student’s *t* test were used. *P* values of <0.05 were considered statistically significant.
