# Supplementary material for: Evaluation of a SARS-CoV-2 Capture IgM Antibody Assay in Convalescent Sera
Source: Microbiol Spectr. 2021 Sep 8;9(2):e00458-21. doi: 10.1128/Spectrum.00458-21 (PMC8557898; doi:10.1128/Spectrum.00458-21)
Supplement: SUPPLEMENTAL FILE 1 — Supplemental material. Download SPECTRUM00458-21_Supp_1_seq7.pdf, PDF file, 0.1 MB [file spectrum00458-21_supp_1_seq7.pdf]

Figure S1: Blow up from Figure 2B of low P-N values for S and N IgG for control specimens.

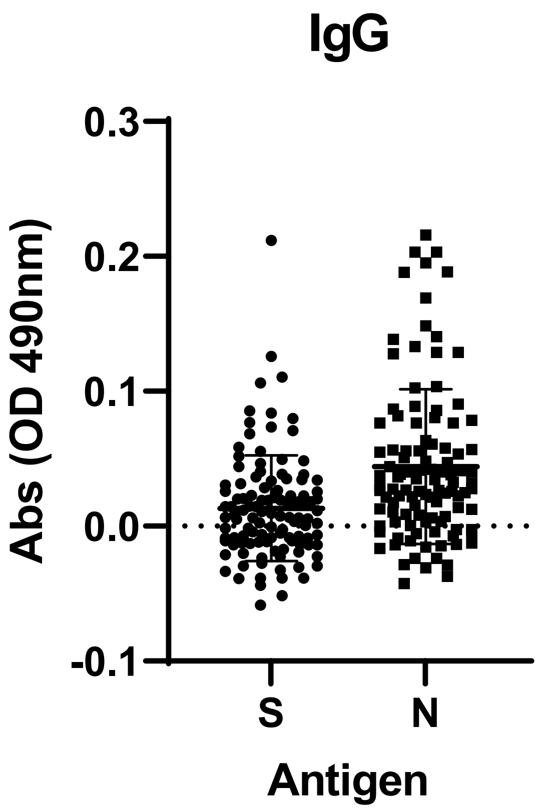

Figure S2: Receiver operating characteristic curves of IgM and IgG assays

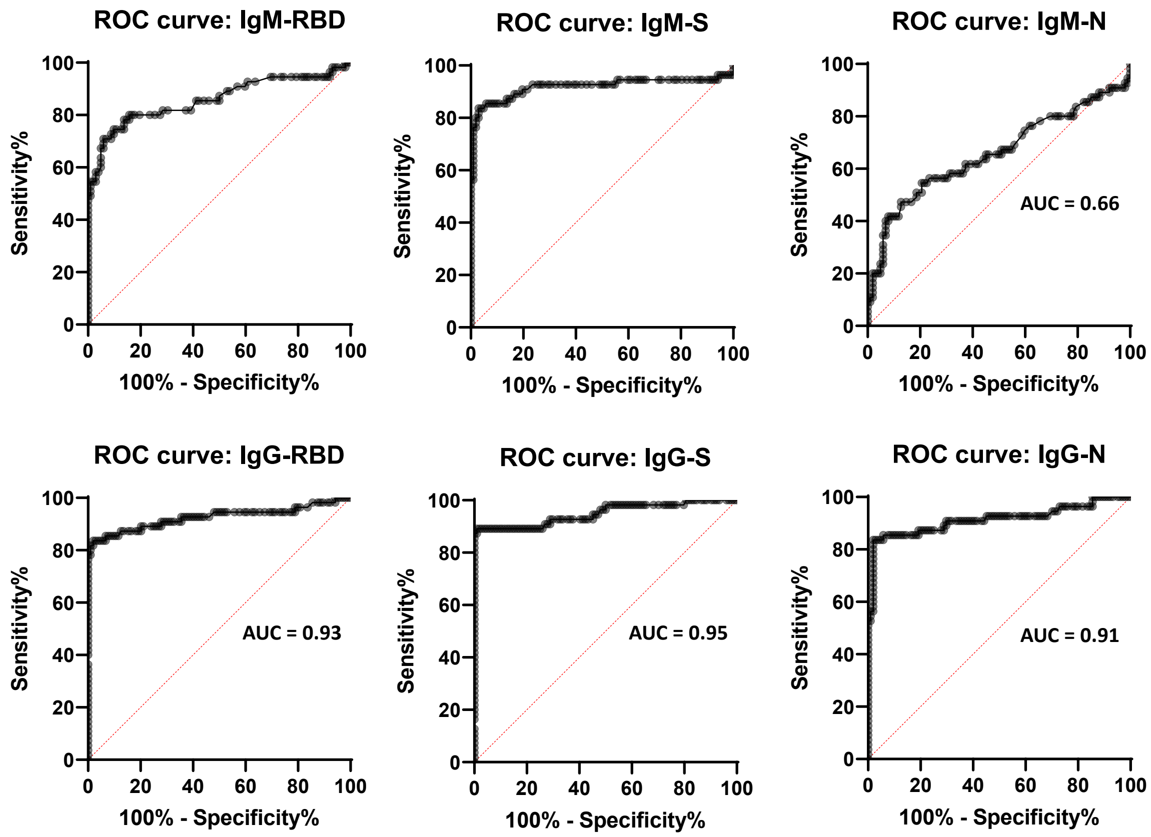

Supplemental Figure Legends

Figure S1: Blow up from Figure 2B of low P-N values for S and N IgG for control specimens.

We considered S IgG P-N values >0.140 and N IgG P-N values >0.190 to be outlier values to be excluded from determination of cutoff values. S: Spike protein, N: Nucleocapsid protein.

Figure S2: Receiver operating characteristic curves of IgM and IgG assays

Absorbance values from controls and COVID-19 PCR+ specimens are graphed for receiver operating characteristic (ROC) curves and the area under the curve (AUC) are calculated with GraphPad software. A) IgM ELISA with RBD, S, and N antigens. B) IgG ELISA with RBD, S, and N antigens. RBD: receptor binding domain, S: spike protein, N: nucleocapsid protein.
